# A unified frame of predicting side effects of drugs by using linear neighborhood similarity

**DOI:** 10.1186/s12918-017-0477-2

**Published:** 2017-12-14

**Authors:** Wen Zhang, Xiang Yue, Feng Liu, Yanlin Chen, Shikui Tu, Xining Zhang

**Affiliations:** 1 School of Computer, Wuhan University, Wuhan, 430072 China; 2 International School of Software, Wuhan University, Wuhan, 430072 China; 3 School of Mathematics and Statistics, Wuhan University, Wuhan, 430072 China; 4 Department of Computer Science and Engineering, Shanghai Jiao Tong University, Shanghai, 200240 China

**Keywords:** Drug side effects, Linear neighborhood similarity, Missing side effects

## Abstract

**Background:**

Drug side effects are one of main concerns in the drug discovery, which gains wide attentions. Investigating drug side effects is of great importance, and the computational prediction can help to guide wet experiments. As far as we known, a great number of computational methods have been proposed for the side effect predictions. The assumption that similar drugs may induce same side effects is usually employed for modeling, and how to calculate the drug-drug similarity is critical in the side effect predictions.

**Results:**

In this paper, we present a novel measure of drug-drug similarity named “linear neighborhood similarity”, which is calculated in a drug feature space by exploring linear neighborhood relationship. Then, we transfer the similarity from the feature space into the side effect space, and predict drug side effects by propagating known side effect information through a similarity-based graph. Under a unified frame based on the linear neighborhood similarity, we propose method “LNSM” and its extension “LNSM-SMI” to predict side effects of new drugs, and propose the method “LNSM-MSE” to predict unobserved side effect of approved drugs.

**Conclusions:**

We evaluate the performances of LNSM and LNSM-SMI in predicting side effects of new drugs, and evaluate the performances of LNSM-MSE in predicting missing side effects of approved drugs. The results demonstrate that the linear neighborhood similarity can improve the performances of side effect prediction, and the linear neighborhood similarity-based methods can outperform existing side effect prediction methods. More importantly, the proposed methods can predict side effects of new drugs as well as unobserved side effects of approved drugs under a unified frame.

## Background

A drug is a chemical substance which can treat, cure or prevent diseases, but all drugs can may have unexpected effects. In this paper, side effects refer to adverse effects of drugs. According to the reports of Food and Drug Administration (FDA), many drugs were withdrawn from markets because of fatal side effects. Identifying side effects of candidate drug molecules is critical for the success of drug discovery [1–6]. For drug safety, the investigation of side effects should be conducted before marketing new drugs. Since wet methods are usually time-consuming and labor-intensive, researchers developed the computational methods to predict drug side effects.

**Fig. 1:**
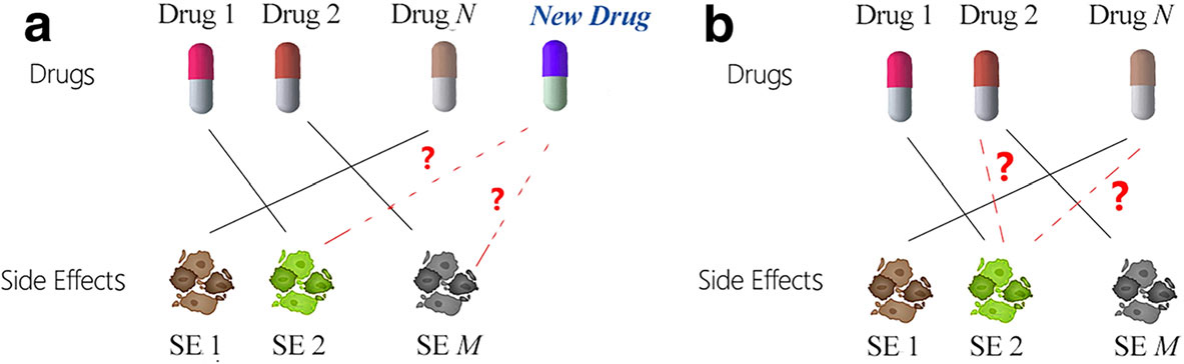
Predicting side effects of new drugs (**a**) and predicting missing side effects of approved drugs (**b**)

For a long time, researchers defined preclinical drug-induced effect patterns to investigate the structure-response relationships or structure-property relationships [7–11], and then utilized them to identify drug side effects. However, these methods have to analyze data case by case, and are not suitable for complicated data. In recent years, the machine learning technique becomes more and more popular, and has been introduced to predict drug side effects. In general, machine learning-based methods are designed to complete two tasks. As demonstrated in Fig. 1, one task is to predict side effects of new drugs (abbreviated “SEND”), and the other task is to predict missing side effects of approved drugs (abbreviated “SEAD”).

As far as we know, many methods have been proposed for the SEND task, and they usually predict drug side effects from their structures or related features. Huang [12] considered drug targets, protein-protein interaction networks and gene ontology annotations, and adopted two types of classifiers: support vector machine (SVM) and logistic regression, and then built prediction models. Pauwels [13] explored chemical substructures of drugs, and utilized k-nearest neighbor classifier, support vector machine, ordinary canonical correlation analysis and sparse canonical correlation analysis to construct prediction models respectively. Yamanishi [14, 15] adopted the sparse canonical correlation analysis to build models based on drug substructures and drug targets. Liu [16] merged five types of drug feature vectors, and respectively utilized logistic regression, naive Bayes, k-nearest neighbor classifier, random forest and SVM to build prediction models. Huang [17] combined protein-protein interaction networks and drug substructures to build prediction models by using SVM. Zhang formulated the side effect prediction as the multi-label learning, and adopted the multi-label KNN to make predictions [18]. There are also several methods designed for the SEAD task. Cheng [19] utilized the resource allocation method to infer missing side effects from the known side effect-based network. Zhang formulated the original problem as the recommender systems, and utilized the resource allocation method, the restricted Boltzmann machine method and the collaborative filtering method to predict unobserved side effects [20]. In general, most existing methods were developed for either SEND task or SEAD task, but few methods can be used for both tasks.

In related studies, researchers usually assumed that similar drugs may induce same side effects, and then built side effect prediction models based on the assumption. The assumption is established on the biological common sense, and similarity-based models have good performances in the side effect prediction. Clearly, the drug-drug similarity is the key to the development of similarity-based models. In previous work [21], we considered a new measure named “linear neighborhood similarity” to calculate drug-drug similarity, and built prediction models to predict side effect of new drugs. In this paper, we present a unified frame based on linear neighborhood similarity to predict side effects of new drugs (SEND task) as well as unobserved side effects of approved drugs (SEAD task).

In this paper, we present the linear neighborhood similarity to calculate drug-drug similarity in a drug feature space, and then transfer the linear neighborhood similarity from the feature space into the side effect space. Therefore, we can predict drug side effects by propagating known side effect information through a similarity-based graph. We propose method “LNSM” and its extension “LNSM-SMI”, which respectively make use of single features and multiple features to predict side effects of new drugs (SEND task); we propose the method “LNSM-MSE” which can predict unobserved side effect of approved drugs based on known side effects (SEAD task). The computational experiments show that the linear neighborhood similarity can produce better performances than other similarity measures in our models. When evaluated by cross validation, the proposed methods can produce high-accuracy performances for both SEND task and SEAD task, and outperform benchmark methods.

## Methods

### Datasets

Motivated by studies on big data, researchers have constructed several databases to facilitate the computational works about drugs. SIDER database [22] contains approved drugs and their reported side effects, which were extracted from public documents and package inserts. PubChem Compound Database [23, 24] contains experimentally validated information about substances, especially their structures. DrugBank database [25–28] contains FDA-approved small molecule drugs, biotech drugs, nutraceuticals, experimental drugs and their related non-redundant protein (drug target, enzyme, transporter, carrier) sequences. KEGG DRUG database [29] is a comprehensive database for approved drugs in Japan, USA, and Europe, providing chemical structures, targets, metabolizing enzymes and etc.

Various features about drugs can be extracted from above databases. The drug chemical substructures provide direct information related with side effects, and are available in PubChem Compound Database. Drug targets may play roles in the particular metabolic or signaling pathway, and thus incur side effects; transporters are responsible for drug absorption, distribution and excretion in tissues; enzymes affect the metabolism to activate drugs, and may be associated with side effects. The pathways and indications are usually considered as the direct factors that induce drug side effects. The information about targets, transporters, enzymes and pathways are available in DrugBank database. Drug indications are provided in SIDER database.

From above data sources, Pauwels et al. [13], Mizutani et al. [14] and Liu et al. [16] compiled several benchmark datasets, and used them for the drug side effect prediction. In our previous work [18], we also compiled a dataset, and we named it “SIDER 4 dataset” [18]. Table 1 detailedly describe above mentioned datasets. The datasets contain drugs and their side effects, and include drug-related features as well. The features in different datasets are introduced. Pauwels’s dataset has only one drug feature: substructure, and Mizutani’s dataset has two features: substructures and targets; both Liu’s dataset and SIDER 4 dataset has six drug-related features. Numbers in Table 1 represent the number of corresponding descriptors for a feature. For example, 881 types of substructures are defined in PubChem, and the feature “substructure” has 881 descriptors because of 881 types of substructures.

**Table 1:** Details about benchmark datasets

Dataset	#drug	#side effect	#substructure	#target	#transporter	#enzyme	#pathway	# indication
Pauwels’s dataset	888	1385	881	N.A	N.A	N.A	N.A	N.A
Mizutani’s dataset	658	1339	881	1368	N.A	N.A	N.A	N.A
Liu’s dataset	832	1385	881	786	72	111	173	869
SIDER 4 dataset	1080	2260	881	1050	96	160	268	2537

*N.A* means unavailable information

### Linear neighborhood similarity

As introduced above, we usually have different features to describe the chemical or biological characteristics of drugs. Since one feature is actually a set of descriptors, a drug can be described by a subset of descriptors in the feature, and thus represented as a binary feature vector, whose dimensions means the presence or absence of descriptors by using the value 1 or 0. When we have different features, we can represent a drug as feature vectors in different feature spaces.

A drug can be considered as a data point in the feature space. How to calculate drug-drug similarity in a feature space is of the most importance for the drug side effect prediction. As far as we know, researcher have proposed several measures to calculate the similarity between data points in the feature space, and popular similarity measures are Jaccard similarity, Cosine similarity and Gauss similarity. Here, we present a novel similarity measure “linear neighborhood similarity” for the side effect prediction, and introduce them as below.

Roweis et al. [30] revealed that the locally linear patch of the manifold in a feature space can be described by data points and neighbor data points; Wang et al. [31] discovered that each point in the high-dimension space may be reconstructed by its neighbors.

Let *X*_*i*_ denote the *p*-dimensional feature vector of drugs *d*_*i*_ in a feature space, *i* = 1, 2, ⋯*N*. By considering feature vectors as data points in the feature space, we assume that a data point *X*_*i*_ approximate to the linear combination of neighbor data points, and write the objective function, which minimizes the reconstruction error,

1 $$ {\displaystyle \begin{array}{l}{\upvarepsilon}_i=\parallel {X}_i-{\sum}_{i_{j,}{X}_{i_j}\in N\left({X}_i\right)}{w}_{i,{i}_j}{X}_{i_j}{\parallel}^2+\uplambda \parallel {w}_i{\parallel}^2\\ {}={\sum}_{X_{i_j}{X}_{i_k}\in N\left({X}_i\right)}{w}_{i,{i}_j}{G}_{i_j,{i}_k}{w}_{i,{i}_k}+\uplambda {\sum}_{X_{i_j}\in N\left({X}_i\right)}{\left({w}_{i,{i}_j}\right)}^2={w_i}^T\left({G}^i+\uplambda \mathrm{I}\right){w}_i\\ {}\mathrm{s}.\mathrm{t}.{\sum}_{X_{i_j}\in N\left({X}_i\right)}{w}_{i,{i}_j}=1,\kern0.5em {w}_{i,{i}_j}\ge 0\end{array}} $$

where *N*(*X*_*i*_) are the set of *K* nearest neighbors of *X*_*i*_. *I* is the identity matrix of order *N*.w_*i*_ = (*w*_*i*, 1_, *w*_*i*, 2_, ⋯, *w*_*i*, *K*_)^*T*^. ${G}^i=\left({G}_{i_j,{i}_k}\right)$. If ${X}_{i_j},{X}_{i_k}\in N\left({X}_i\right),\kern0.5em {G}_{i_j,{i}_k}={\left({X}_i-{X}_{i_j}\right)}^T\left({X}_i-{X}_{i_j}\right)$; otherwise, ${G}_{i_j,{i}_k}=0$; *i*_*j*_ = 1, 2, ⋯, *K*,  *i*_*k*_ = 1, 2, ⋯, *K*. ${w}_{i,{i}_j}$ describe how to construct *X*_*i*_ from ${X}_{i_j}$, and be approximately taken as the similarity between two drugs. The first term of (1) is the reconstruction error; the second term of (1) is for regularization, and λ is the hyper parameter.

The parameter λ is very important for the regularization form of (1). Here, we discuss how to set the parameter. Since ${\sum}_{X_{i_j}\in N\left({X}_i\right)}{w}_{i,{i}_j}$ =1, 0 ≤ ‖*w*_*i*_‖^2^ ≤ 1, and then $\parallel {X}_i-{\sum}_{i_{j,}{X}_{i_j}\in N\left({X}_i\right)}{w}_{i,{i}_j}{X}_{i_j}{\parallel}^2=\parallel {\sum}_{i_{j,}{X}_{i_j}\in N\left({X}_i\right)}{w}_{i,{i}_j}\left({X}_i-{X}_{i_j}\right){\parallel}^2\le p$. *p* is the dimension of feature vectors in the feature space. Clearly, *p* ≫ 1, and we can let λ = 1 to make sure that the error term is greater than the regularization term in (1).

We can adopt the standard quadratic programming technique to solve (1) for each data point *X*_*i*_, *i* = 1, 2, ⋯, *N*. The pairwise similarities between *N* drugs can be written as a *N* × *N* similarity matrix *W* = (*w*_1_, w_2_, ⋯, w_*N*_)^*T*^. We notice that the regularization term is not used if λ = 0. Therefore, we can calculate the linear neighborhood similarity which we name “LN similarity” if λ = 0, and calculate the regularization form of linear neighborhood similarity which we name “RLN similarity” if λ = 1.

**Fig. 2:**
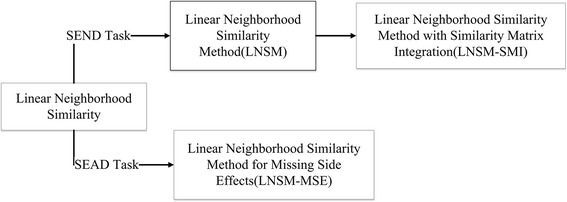
A unified frame of predicting side effects of drugs by using linear neighborhood similarity

By using linear neighborhood similarity, we can develop prediction methods for the SEND task and SEAD task, which are described in Fig. 2. Methods for SEND task are introduced in section 2.3, and the method for SEAD Task is introduced in section 2.4.

### Linear neighborhood similarity-based methods for SEND task

In this section, we propose methods for the SEND task by using the linear neighborhood similarity. One method named “LNSM” is to make predictions based on single features about drugs; the other named “LNSM-SMI” is the extension of LNSM, which can make predictions by integrating multiple features about drugs.

### Linear neighborhood similarity method (LNSM)

Given *N* drugs, these drugs represented as feature vectors *X*_1_, *X*_2_, ⋯, *X*_*N*_ in a *p* -dimensional feature space, where *X*_*i*_ = (*X*_*i*1_, *X*_*i*2_, ⋯, *X*_*ip*_) . Suppose we want to predict *M* types of side effects for drugs, the presence or absence of side effects for *N* drugs can be represented as *M*-dimensional vectors named side effect profiles *Y*_1_, *Y*_2_, ⋯, *Y*_*N*_. ${Y}_i=\left({Y}_{i1},\kern0.5em {Y}_{i2},\kern0.5em \cdots, \kern0.5em {Y}_{iM}\right)$, where *Y*_*ij*_ = 1, if the *i*th drug has the *j*th side effect; else, *Y*_*ij*_ = 0, *i* = 1, 2, ⋯*N*, *j* = 1, 2, ⋯*M*. Therefore, ${\left\{\left({X}_i,{Y}_i\right)\right\}}_{i=1}^N$ are annotated dataset for training models. We respectively concentrate *X*_1_, *X*_2_, ⋯, *X*_*N*_ and *Y*_1_, *Y*_2_, ⋯, *Y*_*N*_ row by row, and obtain two matrices and *Y*. In the feature space, we can easily calculate linear neighborhood similarities between *N* drugs, which are denoted by a similarity matrix *W*. Then, we describe how to build LNSM models.

First of all, we construct a directed graph, which uses *N* given drugs as nodes and drug-drug similarities as edge weights. We consider a side effect term as a type of label, and a node has the label if the drug has the side effect. The *i*th column of *Y* response to the labels for *N* nodes in terms of *i*th side effect term. Label information is propagated on the graph, by following the rule that a node absorbs labels of neighbors with the probability *α* and retain the initial labels with the probability 1 − *α*. Considering all side effect terms simultaneously, we can formulate the update equation in the matrix from,

2 $$ {\mathsf{Y}}^{\mathit{\mathsf{p}}+\mathsf{1}}=\mathit{\mathsf{\alpha W}}{\mathsf{Y}}^{\mathit{\mathsf{p}}}+\left(\mathsf{1}-\mathit{\mathsf{\alpha}}\right){\mathsf{Y}}^{\mathsf{0}} $$

Where Y^0^ is the matrix for initial label information, and Y = Y^0^. *Y* is matrix representing the updated labels for $\mathit{\mathsf{N}}$ nodes. The iteration will converge to

3 $$ {\mathit{\mathsf{Y}}}^{\prime }=\left(\mathsf{1}-\mathit{\mathsf{\alpha}}\right){\left(\mathit{\mathsf{I}}-\mathit{\mathsf{\alpha W}}\right)}^{-\mathsf{1}}{\mathit{\mathsf{Y}}}^{\mathsf{0}} $$

Where *I* is the identity matrix of order *N*. *Y* ^′^ is final labels for $\mathit{\mathsf{N}}$ nodes.

When we have a new drug *X*_*new*_ for prediction, we take the drug as out-of-sample data, and calculate the similarities between *X*_*new*_ and *N* known drugs in the feature space. The similarities are represented by a vector *W*_*new*_ = (*w*_*new*, 1_, *w*_*new*, 2_, ⋯, *w*_*new*, *N*_). Thus, we can predict the side effects of *X*_*new*_,

$$ {\mathit{\mathsf{Y}}}_{\mathit{\mathsf{new}}=}{\mathit{\mathsf{W}}}_{\mathit{\mathsf{new}}}\times \mathit{\mathsf{Y}} $$

According to the above discussion, LNSM predicts side effect of new drugs from single drug features.

### Linear neighborhood similarity method with similarity matrix integration

In order to predict side effects of new drugs, researchers usually collect various drug features, and construct the relationship between features and their side effects. When we have multiple drug features, we have to face the challenges of integrating features to make predictions. For the purpose, we propose the linear neighborhood similarity method with similarity matrix integration (LNSM-SMI) by extending LNSM.

Given *N* drugs, we have *K* features to describe characteristics of drugs. Let ${X}_i^k$ denote the feature vector based on *k*th feature for the *i*th drug, and *Y*_*i*_ denotes the side effect vector for the *i*th drug. In *K* feature spaces, we calculate similarities between *N* drugs, and represent them as similarity matrices. *K* features can produce *K* similarity matrices *W*_1_, *W*_2_, ⋯, *W*_*K*_. Then, we describe how to build models based on multiple features.

First of all, the study in [31] proved the label propagation on the graph shown in (2) is equivalent to a convex optimization problem,

4 $$ {\mathit{\mathsf{\min}}}_{\mathit{\mathsf{Y}}}\mathit{\mathsf{\alpha}}\left(\mathit{\mathsf{tr}}\left({\mathit{\mathsf{Y}}}^{\mathit{\mathsf{T}}}\left(\mathit{\mathsf{I}}-\mathit{\mathsf{W}}\right)\mathit{\mathsf{Y}}\right)\right)+\left(\mathsf{1}-\mathit{\mathsf{\alpha}}\right){\left\Vert \mathit{\mathsf{Y}}-{\mathit{\mathsf{Y}}}^{\mathsf{0}}\right\Vert}_{\mathit{\mathsf{F}}}^{\mathsf{2}} $$

When we have similarity matrices *W*_1_, *W*_2_, ⋯, *W*_*K*_ based on *K* features, we consider the linear sum of these matrices $\sum \limits_{i=1}^K{\theta}_i{W}_i$. By replacing *W* in (4) with $\sum \limits_{i=1}^K{\theta}_i{W}_i$, we can obtain the optimization problem,

5 $$ \underset{\mathit{\mathsf{Y}},\mathit{\mathsf{\theta}}}{\mathit{\mathsf{\min}}}\ \mathit{\mathsf{\alpha}}\left(\mathit{\mathsf{tr}}\left({\mathit{\mathsf{Y}}}^{\mathit{\mathsf{T}}}\sum \limits_{\mathit{\mathsf{i}}=\mathsf{1}}^{\mathit{\mathsf{K}}}{\mathit{\mathsf{\theta}}}_{\mathit{\mathsf{i}}}\left(\mathit{\mathsf{I}}-{\mathit{\mathsf{W}}}_{\mathit{\mathsf{i}}}\right)\mathit{\mathsf{Y}}\right)\right)+\left(\mathsf{1}-\mathit{\mathsf{\alpha}}\right){\left\Vert \mathit{\mathsf{Y}}-{\mathit{\mathsf{Y}}}^{\mathsf{0}}\right\Vert}_{\mathit{\mathsf{F}}}^{\mathsf{2}}+\mathit{\mathsf{\delta}}{\left\Vert \mathit{\mathsf{\theta}}\right\Vert}^{\mathsf{2}} $$

$$ \mathit{\mathsf{s}}.\mathit{\mathsf{t}}.\sum \limits_{\mathit{\mathsf{i}}=\mathsf{1}}^{\mathit{\mathsf{K}}}{\mathit{\mathsf{\theta}}}_{\mathit{\mathsf{i}}}=\mathsf{1};\forall \mathit{\mathsf{i}},{\mathit{\mathsf{\theta}}}_{\mathit{\mathsf{i}}}\ge \mathsf{0} $$

where *δ*(>0) is hyper parameter for the regularization term ‖*θ*‖^2^.

The matrix *Y* ^0^ = [*Y*_1_, *Y*_2_, ⋯, *Y*_*N*_]^*T*^ represents observed side effects for *N* known drugs, and we can set *Y* = *Y* ^0^ and rewrite (5) as

6 $$ \underset{\mathit{\mathsf{\theta}}}{\mathit{\mathsf{\min}}}\mathit{\mathsf{\alpha}}\left(\mathit{\mathsf{tr}}\left({\left({\mathit{\mathsf{Y}}}^{\mathsf{0}}\right)}^{\mathit{\mathsf{T}}}\sum \limits_{\mathit{\mathsf{i}}=\mathsf{1}}^{\mathit{\mathsf{K}}}{\mathit{\mathsf{\theta}}}_{\mathit{\mathsf{i}}}\left(\mathit{\mathsf{I}}-{\mathit{\mathsf{W}}}_{\mathit{\mathsf{i}}}\right){\mathit{\mathsf{Y}}}^{\mathsf{0}}\right)\right)+\mathit{\mathsf{\delta}}{\left\Vert \mathit{\mathsf{\theta}}\right\Vert}^{\mathsf{2}} $$

$$ \mathit{\mathsf{s}}.\mathit{\mathsf{t}}.\sum \limits_{\mathit{\mathsf{i}}=\mathsf{1}}^{\mathit{\mathsf{K}}}{\mathit{\mathsf{\theta}}}_{\mathit{\mathsf{i}}}=\mathsf{1};\forall \mathit{\mathsf{i}},{\mathit{\mathsf{\theta}}}_{\mathit{\mathsf{i}}}\ge \mathsf{0} $$

We introduce the Lagrange Multiplier terms *λ* and *η* = (*η*_1_, *η*_2_, ⋯, *η*_*K*_)^*T*^ to solve the optimization problem,

7 $$ \mathit{\mathsf{L}}\left(\mathit{\mathsf{\theta}},\mathit{\mathsf{\lambda}},\mathit{\mathsf{\eta}}\right)=\mathit{\mathsf{\delta}}{\mathit{\mathsf{\theta}}}^{\mathit{\mathsf{T}}}\mathit{\mathsf{\theta}}+\mathit{\mathsf{\alpha}}{\mathit{\mathsf{C}}}^{\mathit{\mathsf{T}}}\mathit{\mathsf{\theta}}-\mathit{\mathsf{\lambda}}\left({\mathit{\mathsf{e}}}^{\mathit{\mathsf{T}}}\mathit{\mathsf{\theta}}-\mathsf{1}\right)-{\mathit{\mathsf{\eta}}}^{\mathit{\mathsf{T}}} $$

Where *c*_*i*_ = *trace*((*Y* ^0^)^*T*^(*I* − *W*_*i*_)*Y* ^0^), *C* = (*c*_1_, *c*_2_, ⋯, *c*_*K*_)^*T*^, and *e* = (1, 1, ⋯, 1)^*T*^. The KKT condition is,

8 $$ \left\{\begin{array}{c}{\nabla}_{\mathit{\mathsf{\theta}}}\mathit{\mathsf{L}}\left(\mathit{\mathsf{\theta}},\mathit{\mathsf{\lambda}},\mathit{\mathsf{\eta}}\right)=\mathsf{2}\mathit{\mathsf{\delta \theta }}+\mathit{\mathsf{\alpha C}}-\mathit{\mathsf{\lambda e}}-\mathit{\mathsf{\eta}}=\mathsf{0}\\ {}{\mathit{\mathsf{e}}}^{\mathit{\mathsf{T}}}\mathit{\mathsf{\theta}}-\mathsf{1}=\mathsf{0}\\ {}{\mathit{\mathsf{\eta}}}_{\mathit{\mathsf{i}}}\ge \mathsf{0},{\mathit{\mathsf{\theta}}}_{\mathit{\mathsf{i}}}\ge \mathsf{0},{\mathit{\mathsf{\eta}}}_{\mathit{\mathsf{i}}}{\mathit{\mathsf{\theta}}}_{\mathit{\mathsf{i}}}=\mathsf{0},\mathit{\mathsf{i}}=\mathsf{1},\cdots, \mathit{\mathsf{K}}\ \end{array}\kern1em \right. $$

In (8), *L*(*α*, *λ*, *η*) = 2*δθ*_*i*_ + *αc*_*i*_ − *λ* − *η*_*i*_ = 0 and *η*_*i*_ = 2*δθ*_*i*_ + *αc*_*i*_ − *λ*, and thus we know that *θ*_*i*_(2*δθ*_*i*_ + *αc*_*i*_ − *λ*) = 0. Since 0 ≤ *θ*_*i*_ ≤ 1, we can know that *θ*_*i*_ = 0 if *λ* − *αc*_*i*_ ≤ 0; otherwise, *θ*_*i*_ = (*λ* − *αc*_*i*_)/(2*δ*). We reorder *c*_1_, *c*_2_, ⋯, *c*_*K*_ as *c*_1_ ≤ *c*_2_ ≤ ⋯ ≤ *c*_*K*_, and then the corresponding weights *θ*_1_ ≥ *θ*_2_ ≥ ⋯ ≥ *θ*_*l*_ > *θ*_*l* + 1_ = ⋯ = *θ*_*K*_ = 0. Therefore, we can obtain the solution for the optimization problem in (5),

9 $$ \left\{\begin{array}{c}\mathit{\mathsf{l}}=\mathit{\mathsf{\max}}\left\{\mathit{\mathsf{n}}\ \right|\mathit{\mathsf{\delta}}\ge \frac{\mathit{\mathsf{\alpha}}}{\mathsf{2}}{\sum}_{\mathit{\mathsf{k}}=\mathsf{1}}^{\mathit{\mathsf{n}}}\left({\mathit{\mathsf{c}}}_{\mathit{\mathsf{k}}}-{\mathit{\mathsf{c}}}_{\mathit{\mathsf{i}}}\right),\mathit{\mathsf{n}}=\mathsf{1},\mathsf{2}\cdots, \mathit{\mathsf{K}}\Big\}\\ {}\mathit{\mathsf{\lambda}}=\frac{\mathsf{1}}{\mathit{\mathsf{l}}}\left(\mathsf{2}\mathit{\mathsf{\delta}}+\mathit{\mathsf{\alpha}}{\sum}_{\mathit{\mathsf{k}}=\mathsf{1}}^{\mathit{\mathsf{l}}}{\mathit{\mathsf{c}}}_{\mathit{\mathsf{i}}}\right)\\ {}{\mathit{\mathsf{\theta}}}_{\mathit{\mathsf{i}}}=\frac{\mathsf{2}\mathit{\mathsf{\delta}}+\mathit{\mathsf{\alpha}}{\sum}_{\mathit{\mathsf{k}}=\mathsf{1}}^{\mathit{\mathsf{l}}}\left({\mathit{\mathsf{c}}}_{\mathit{\mathsf{k}}}-{\mathit{\mathsf{c}}}_{\mathit{\mathsf{i}}}\right)}{\mathsf{2}\mathit{\mathsf{l}\mathsf{\delta }}},{\mathit{\mathsf{\eta}}}_{\mathit{\mathsf{i}}}=\mathsf{0},\mathit{\mathsf{i}}=\mathsf{1},\cdots, \mathit{\mathsf{l}}\\ {}{\mathit{\mathsf{\theta}}}_{\mathit{\mathsf{i}}}=\mathsf{0},{\mathit{\mathsf{\eta}}}_{\mathit{\mathsf{i}}}=\frac{\mathsf{1}}{\mathit{\mathsf{l}}}\left(\mathit{\mathsf{\alpha}}{\sum}_{\mathit{\mathsf{k}}=\mathsf{1}}^{\mathit{\mathsf{l}}}\left({\mathit{\mathsf{c}}}_{\mathit{\mathsf{i}}}-{\mathit{\mathsf{c}}}_{\mathit{\mathsf{k}}}\right)-\mathsf{2}\mathit{\mathsf{\delta}}\right),\mathit{\mathsf{i}}=\mathit{\mathsf{l}}+\mathsf{1},,\cdots, \mathit{\mathsf{K}}\end{array}\right. $$

Let *c*_*max*_ = max {*c*_1_, *c*_2_, ⋯, *c*_*K*_}. Clearly, the free parameter *δ* determine the number of nonzero weights. In order to guarantee $\delta \ge \frac{\alpha }{2}{\sum}_{k=1}^n\left({c}_k-{c}_i\right)$, we can set $\delta =\frac{\alpha }{2}{\sum}_{k=1}^K\left({c}_{max}-{c}_k\right)$. Therefore, we can estimate weights in a simple form,

10 $$ {\theta}_i=\frac{c_{max}-{c}_i}{\sum_{k=1}^K\left({c}_{max}-{c}_k\right)},i=1,2,\cdots, K $$

When we have a new drug *X*_*new*_ described by *K* features, we can calculate similarities between the new drug *X*_*new*_ and known drugs, represented by *K* vectors ${W}_{new}^i$, *i* = 1, 2, ⋯, *K*. Thus, we can predict the side effects of *X*_*new*_ based on *K* features,

$$ {\mathit{\mathsf{Y}}}_{\mathit{\mathsf{new}}}=\left({\sum}_{\mathit{\mathsf{i}}=\mathsf{1}}^{\mathit{\mathsf{K}}}{\mathit{\mathsf{\theta}}}_{\mathit{\mathsf{i}}}{\mathit{\mathsf{W}}}_{\mathit{\mathsf{new}}}^{\mathit{\mathsf{i}}}\right){\mathit{\mathsf{Y}}}^{\mathsf{0}} $$

Clearly, LNSM-SMI is the extension of LNSM to make use of multiple features for prediction.

### Linear neighborhood similarity method for SEAD task

In this section, we propose the method “LNSM-MSE” to predict missing or unobserved side effects of approved drugs by using the linear neighborhood similarity.

**Fig. 3:**
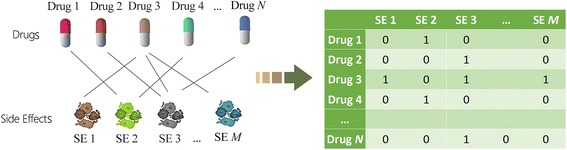
Drug association profiles defined on known side effects

Given *N* Drugs and *M* side effect terms, we known that these drugs have observed side effects. By linking drugs and induced side effects, relations between drugs and induced side effects can be formulated as a bipartite network. The bipartite network can be described by an *N* × *M* association matrix *A*, where *A*_*ij*_ = 1 if the drug *i* induces side effect *j* and *A*_ij_ = 0 otherwise. For each drug *d*_*i*_, i = 1, 2, ⋯, *N*, the associate profile of *d*_*i*_ is the vector *A*(*i*, :) = (*A*_*i*1_, *A*_*i*2_, ⋯, *A*_*iM*_), which represents the known side effects of the drug. The drug-side effect bipartite network and the association matrix are demonstrated in Fig. 3.

Then, we calculate linear neighborhood similarities *W* between drugs based on their association profiles, and construct the directed graph which uses drugs as nodes and use similarities as edge weights. The known side effect information is propagated on the graph as described in section 2.2.2, and the update will converge. Thus, we can predict missing side effects of *N* approved drugs,

$$ \mathit{\mathsf{Y}}=\left(\mathsf{1}-\mathit{\mathsf{\alpha}}\right){\left(\mathit{\mathsf{I}}-\mathit{\mathsf{\alpha W}}\right)}^{-\mathsf{1}}\mathit{\mathsf{A}} $$

If *A*_ij_ = 0, the entry *Y*_*ij*_ indicates the probability of drug *d*_*i*_ inducing the *j*th side effect. Therefore, LNSM-MSE predict missing side effects of approved drugs based on their known side effects.

## Results and discussion

### Evaluation metrics

In the paper, we evaluate prediction models by using five-fold cross validation (5-CV). The five-fold cross validation in the SEND task randomly splits all drugs into equal-sized subsets. In each fold, four subsets of drugs are used as the training set, and other drugs are used as the testing set. The models are constructed on training set with annotated features and side effects, and then predict side effects of drugs in the testing test from features. In the SEAD task, the five-fold cross validation splits all known side effects into equal-sized subsets. We construct the prediction models based on all drugs and known side effects in the training set, and apply the model to predict unobserved side effects for all drugs.

In the SEND task, the side effect prediction is a multi-label learning task [18]. Therefore, we adopt several evaluation metrics for the multi-label classification to evaluate models, i.e. Hamming loss, one-error, coverage, ranking loss and average precision. In addition, we use the area under ROC curve (AUC) and the area under the precision-recall curve (AUPR). The smaller scores of one-error, coverage, ranking loss and hamming loss indicate better results, and the smaller scores of AUC and AUPR mean better results.

For the SEAD task, we adopt several binary classification metrics to evaluate the performances of models, including specificity (SP), sensitivity (SN), accuracy (ACC), F-measure (F), recall, precision, AUC and AUPR,

For all drugs and all side effect terms, the associated drug-side effect pairs which indicate that drug induces the side effect are much more than other pairs. Since data is imbalanced, we adopt the AUPR as the primary metric to evaluate the models in both SEND task and SEAD task.

### Performances of linear neighborhood similarity methods for SEND task

By using the linear neighborhood similarity, we present the linear neighborhood similarity method (LNSM) and the linear neighborhood similarity method with similarity matrix integration (LNSM-SMI). LNSM uses single drug features to make predictions; as the extension of LNSM, LNSM-SMI integrates multiple features for predictions. In this section, we evaluate LNSM and LNSM-SMI based on Liu’s dataset.

### Performances of LNSM

LNSM can build the prediction models based on the single features. Liu’s dataset has a variety of features, and we respectively construct prediction models based on the different features, and evaluate their usefulness.

**Fig. 4:**
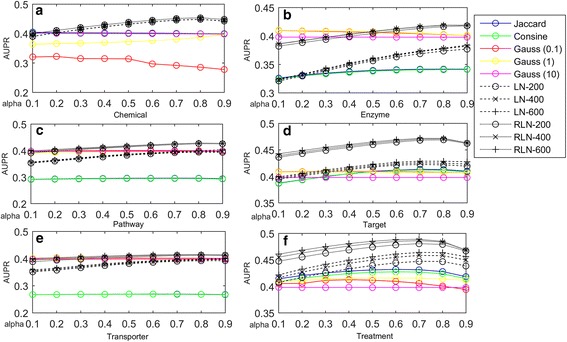
AUPR scores of different similarity-based models based on Liu’s data under different conditions. **a**~**f** demonstrate the performances of models based on different drug features in Liu’s data. LN-200: the models based on the LN similarity and 200 neighbors

LNSM calculates drug-drug similarity in a feature space, and then predict side effects of new drugs. There are two parameters in LNSM: the absorbing probability α and the neighbor number *K*. Liu’s dataset has 832 drugs, and thus the five-fold cross validation has about 665 training drugs in each fold. Therefore, the neighbor number *K* should be less than 665 in our study. To test the impact of parameters on LNSM, we consider α in {0.1,0.2, ⋯0.9} and *K* in {200,400,600} to build prediction models. In addition, we consider different similarities: Jaccard similarity, Cosine similarity and Gauss similarity to compare with the linear neighborhood similarity (LN) and regularized linear neighborhood similarity (RLN). Figure 4 demonstrates AUPR scores of all prediction models evaluated by five-fold cross validation.

According to the results in Fig. 4, LNSM prediction models which use LN similarity and RLN similarity produce robust results for the parameters: the neighbor number *K* and absorbing probability α. RLN similarity is the LN similarity with the regularization term. The introduction of the regularization term usually enhances generalization capability of prediction models. One drawback of LN is that the *G* ^*i*^ in the Eq. (1) may be a singular matrix, and the introduction of the regularization term can alleviate the singular matrix problem in solving quadratic programming. Therefore, we have observed that LNSM models based on RLN similarity can lead to better experimental results than LNSM models based on LN similarity under all conditions. In general, the LNSM models produce the best results when using 400 neighbors and α of 0.8.

Figure 4 also demonstrates the results of prediction models based on different similarities. In fact, the linear neighborhood similarity and its regularized form calculate the similarity in a feature space by considering linear relationship of data points, and the similarity can be transferred into the side effect space and be used by the label propagation, which is also in a linear form. In contrast, other similarities (Jaccard similarity, Cosine similarity and Gauss similarity) calculates the drug-dug similarity in a nonlinear from. Therefore, the models based on LN similarity and RLN similarity yield better AUPR scores than models based on other similarities.

Superiority of LNSM is demonstrated in this section. The parameters: the neighbor number of 400 and α of 0.8 are used for LNSM in the following experiments.

### Performances of LNSM-SMI

When diverse features are available, researchers usually combine or integrate multiple features in order to achieve high-accuracy prediction models [18, 20, 32–38]. As discussed above, we have multiple features to describe chemical and biological characteristics of drugs. Here, we test the performances of the integration method: the linear neighborhood similarity method with similarity matrix integration (LNSM-SMI), which integrate diverse and multiple features.

All prediction models are evaluated based on Liu’s dataset by using 5-fold cross validation. Table 2 shows the performances of integration models LNSM-SMI which use multiple features and LNSM models based on single features. We respectively build six LNSM models by using six features, and build a LNSM-SMI model by integrating six features. As shown in Table 2, the feature “indication” can produce the LNSM model with best performances, and the performances of targets, substructures, pathways, enzymes and transporters are sorted descendingly. Clearly, the data integration model LNSM-SMI can greatly improve the performances of LNSM based on indications, achieving the AUPR scores of 0.5053. The improvements in terms of other evaluation metrics can be observed as well. Therefore, LNSM-SMI can effectively combine multiple features to predict side effects of new drugs.

**Table 2:** 5-CV performances of prediction models on Liu’s dataset

Data	Methods	AUC	AUPR	Hamming Loss	Ranking Loss	One Error	Coverage	Average Precision
Enzyme	LNSM	0.8898	0.4187	0.0473	0.0821	0.1659	846.3846	0.4696
Pathway	LNSM	0.8886	0.4273	0.0470	0.0776	0.1647	814.6298	0.4932
Target	LNSM	0.8991	0.4708	0.0452	0.0690	0.1538	792.3726	0.5216
Transporter	LNSM	0.8896	0.4147	0.0477	0.0817	0.1611	849.3161	0.4762
Treatment	LNSM	0.9013	0.4836	0.0446	0.0710	0.1262	806.8558	0.5232
Substructure	LNSM	0.8944	0.4538	0.0459	0.0714	0.1490	803.5228	0.5184
All data	LNSM-SMI	0.8986	0.5053	0.0435	0.0670	0.1154	789.8486	0.5476

LNSM-SMI has the weights *α*_1_, *α*_2_, ⋯, *α*_*K*_ for similarity matrices, which are calculated from *K* different features. We analyzed how to estimate weights in LNSM-SMI, and give out the analytical solutions in (10). Thus, we investigate weights *α*_1_, *α*_2_, ⋯, *α*_*K*_ in LNSM-SMI models. The weights *α*_1_, *α*_2_, ⋯, *α*_*K*_ directly indicate the features’ contributions to the data integration models, and we can observe that features which have better performances in LNSM can usually gain greater weights in LNSM-SMI. We further conduct simulation experiments to demonstrate the importance of weights in LNSM-SMI. Here, we randomly generate 100 sets of weights, and use them to construct LNSM-SMI models. We analyze the AUPR scores of these LNSM-SMI models evaluated by 5-CV, and our statistics is 0.4912 ± 0.0104. The results show that the optimal weights are very important for LNSM-SMI, and arbitrary weights cannot yield the superior performances. Clearly, our estimation in (10) can effectively determine the optimal weights, and produce the satisfying results in the computational experiments,

### Performances of LNSM-MSE for SEAD task

By using the linear neighborhood similarity, we develop LNSM-MSE to predict missing side effect of approved drugs.

**Fig. 5:**
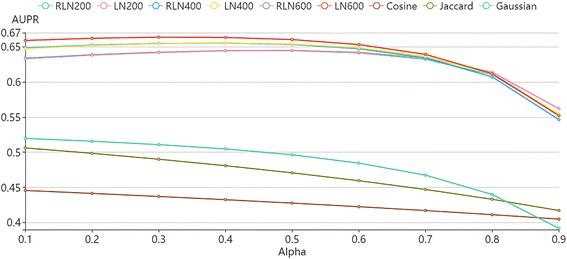
AUPR scores of LNSM-MSE models using different similarities for SEAD task

LNSM-MSE calculates drug-drug similarity based on the drug side effect association profiles, which are defined on the known side effects of approved drugs, and then build models. First of all, we consider different similarity measures, including Jaccard similarity, Cosine similarity, Gauss similarity, LN similarity and RLN similarity for the purpose of comparison. We consider the neighbor number *K* 200,400 and 600 for LN similarity and RLN similarity. The probability α 0.1, 0.2, 0.3, 0.4, 0.5, 0.6, 0.7, 0.8 and 0.9 are considered for the label propagation. Figure 5 demonstrates AUPR scores of different models. The results indicate that LN similarity and RLN similarity also outperform other similarities in predicting missing side effects of approved drugs (SEAD task). Since LN similarity has similar performances as RLN similarity in the SEAD task, we use RLN similarity to construct LNSM-MSE in the following study.

**Fig. 6:**
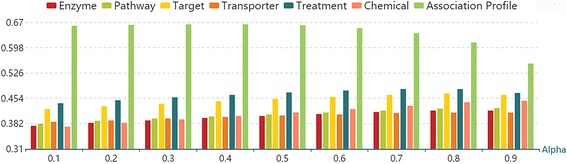
AUPR scores of models based on different features for SEAD task (neighbor number = 600)

Since we have multiple features about drugs in Liu’s dataset, we can calculate drug-drug RLN similarities based on different features, and build the prediction models which are similar to LNSM-MSE. Here, we respectively use different features to construct LNSM-MSE models, and compare different features. As shown in Fig. 6, the results demonstrate that the association profile can have significantly better performances than other features. Clearly, association profiles of drugs can bring critical information for modelling, and LNSM-MSE can produce the AUPR score greater than 0.65 by only using the association profile.

**Fig. 7:**
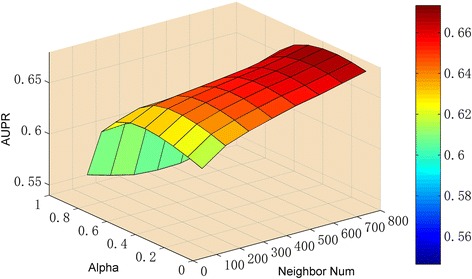
The visualization of parameters and AUPR scores of LNSM-MSE

Finally, we consider greater ranges for parameters neighbor number *K* and the absorbing probability α, and determine the optimal parameters for the LNSM-MSE, which utilizes the association profile and RLN similarity. For neighbor number *K*, we consider 100, 200, …., 800; we consider the absorbing probability α 0.1,0.2,…0.9. We try different parameter combinations, and AUPR scores of LNSM-MSE models based on different parameter values are visualized in Fig. 7. LNSM-MSE can produce the best results when K = 800 and *α* = 0.3, and these parameter values are used for final LNSM-MSE models in following experiments.

### Comparison with benchmark methods

As we mentioned, lots of methods have been proposed to predict drug side effects, and some methods which provided source codes and datasets are usually used as benchmark methods for comparison. These benchmark methods we consider are Pauwels’s method [13], Mizutani’s method [14], Cheng’s method [19], Liu’s method [16], RBMBM [20], INBM [20] and FS-MLKNN [18]. In this paper, we present a unified frame to handle two side effect prediction tasks by using linear neighborhood similarity. However, these benchmark methods are usually designed for either SEND task [13, 14, 18] or SEAD task [19, 20]; only on method: Liu’s method is suitable for both tasks. Therefore, we compare our proposed methods with benchmark methods respectively in two tasks.

### Comparison with benchmark methods for SEND task

For the SEND task, we adopt Pauwels’s method, Liu’s method, Mizutani’s method and FS-MLKNN as benchmark methods for comparison. We replicate these methods by using their publicly available source codes or following details in publications. We respectively construct our prediction models by using the same datasets which were ever used for benchmark methods. Since only one feature “substructure” in Pauwels’s dataset and Mizutani’s dataset was usually used for modeling, we build LNSM models on these datasets to compare with corresponding methods. Liu’s dataset has multiple features, and were ever used by Liu’s method and FS-MLKNN, and thus we build LNSM-SMI models based on multiple features to make the comparison. Table 3 shows results of all methods evaluated by 5-fold cross validation. Clearly, the proposed methods outperform benchmark methods under the same experimental conditions.

**Table 3:** Performances of our methods and other state-of-the-art methods

Dataset	Method	AUC	AUPR	Hamming Loss	Ranking Loss	One Error	Coverage	Average Precision
Pauwels’s dataset	Pauwels’s method	0.8827	0.3883	0.0577	0.0827	0.1779	832.7827	0.4616
	LNSM	0.8941	0.4491	0.0444	0.0713	0.1633	790.9471	0.5126
Mizutani’s dataset	Mizutani’s method	0.8665	0.4107	0.0557	0.0888	0.1854	862.9757	0.4795
	LNSM	0.8946	0.4624	0.0499	0.0746	0.1581	805.8875	0.5170
Liu’s dataset	Liu’s method	0.8850	0.2514	0.0721	0.0927	0.9291	837.4579	0.2610
	FS-MLKNN	0.9034	0.4802	0.0524	0.0703	0.1202	795.9435	0.5134
	LNSM-SMI	0.8986	0.5053	0.0435	0.0670	0.1154	789.8486	0.5476

We further implement the independent experiments to evaluate the practical capability of our methods. Here, we adopt Liu’s method and FS-MLKNN for comparison, for they usually have good performances on different datasets. The SIDER 4 dataset covers 1080 drugs, which have 771 drugs overlapped with Liu’s dataset and 309 newly added drugs. In independent experiments, we train prediction models based on 771 drugs, and then make prediction for 309 new drugs. Table 4 demonstrates results of all models, and LNSM-SMI has significant advantages on the AUPR scores.

**Table 4:** Performances of different methods in the independent test

Method	AUC	AUPR	Hamming Loss	Ranking Loss	One Error	Coverage	Average Precision
Liu’s method	0.8772	0.1766	0.0421	0.1150	0.9870	1587.5663	0.1816
FS-MLKNN	0.8722	0.3109	0.0373	0.1038	0.1851	1535.9223	0.3649
LNSM-SMI	0.8786	0.3465	0.0291	0.0969	0.2013	1488.2977	0.3906

For each testing drug, we respectively consider top 100 and top 200 predicted side effect terms, and investigate how much known side effects can be found out. We calculate recall scores for drugs one by one, and conduct statistics on the results. By evaluating top 100 predictions, the statistics on AUPR scores of Liu’s method, FS-MLKNN and LNSM-SMI are 0.4161 ± 0.0239, 0.5157 ± 0.0293, 0.5421 ± 0.0334; the statistics in evaluating top 200 predictions are 0.6261 ± 0.0262, 0.6605 ± 0.0263, 0.6840 ± 0.0285. LNSM-SMI has identified about 54% known side effects on average when checking up top 100 predicted side effects out of 2260 side effect terms, and has identified about 68% known side effects on average when checking up top 200 predictions. Therefore, LNSM-SMI is effective for predicting side effect of new drugs.

### Comparison with benchmark methods for SEAD task

We propose LNSM-MSE to predict missing side effects of approved drugs from known side effects. In predicting missing side effects of approved drugs, we adopt Cheng’s method [19], Liu’s method [16], INBM [20] and RBMBM [20] for comparison. Liu’s method makes use of multiple features for predictions, and other methods only use the known side effects to predict missing ones. Therefore, we construct LNSM-MSE and benchmark methods on benchmark datasets, and use 5-fold cross validation to evaluate models.

The performances of all methods are shown in Table 5. Clearly, LNSM-MSE can outperform the benchmark methods on the benchmark datasets, and significantly improve the AUPR score from 0.64 to 0.67. Moreover, LNSM-MSE has the better performances in terms of other evaluation metrics. Therefore, LNSM-MSE is useful and suitable for the SEAD Task.

**Table 5:** Performances of LNSM-MSE and benchmark methods evaluated by 5-CV

Dataset	Methods	AUPR	AUC	SN	SP	Precision	Accuracy	F
Pauwels’s dataset	Liu’s method	0.345	0.920	0.643	0.950	0.400	0.934	0.493
	Cheng’s method	0.588	0.922	0.587	0.975	0.547	0.955	0.566
	RBMBM	0.612	0.941	0.605	0.977	0.579	0.958	0.592
	INBM	0.641	0.934	0.608	0.979	0.605	0.961	0.607
	LNSM-MSE	0.671	0.948	0.629	0.980	0.625	0.963	0.627
Mizutani’s dataset	Liu’s method	0.366	0.918	0.637	0.948	0.418	0.930	0.505
	Cheng’s method	0.599	0.923	0.593	0.973	0.560	0.951	0.576
	RBMBM	0.619	0.939	0.614	0.974	0.581	0.954	0.597
	INBM	0.646	0.932	0.616	0.976	0.605	0.956	0.611
	LNSM-MSE	0.676	0.944	0.627	0.979	0.635	0.959	0.631
Liu’s dataset	Liu’s method	0.278	0.907	0.669	0.930	0.341	0.917	0.452
	Cheng’s method	0.592	0.922	0.589	0.974	0.550	0.954	0.569
	RBMBM	0.616	0.941	0.608	0.976	0.581	0.957	0.594
	INBM	0.641	0.934	0.607	0.979	0.606	0.959	0.606
	LNSM-MSE	0.673	0.948	0.631	0.979	0.624	0.962	0.628

## Conclusions

This paper presents a novel similarity measure named “linear neighborhood similarity” to calculate drug-drug similarity, and develop a unified frame of predicting side effects of new drugs (SEAD task) as well as missing side effects of approved drugs (SEND task). Therefore, we propose the method “LNSM” and its extension “LNSM-SMI” to predict the side effects of new drugs; we propose the method “LNSM-MSE” to predict missing side effects of approved drugs. In computational experiments, proposed methods can produce good results, and outperform benchmark methods in two tasks. The proposed methods have great potential in predicting drug side effects.

## Abbreviations

5-CV: 5-fold cross validation
AUC: area under ROC curve
AUPR: area under precision-recall curve
SEAD: predicting missing side effect of approved drug
SEND: predicting side effects of new drugs
